# Therapeutic Efficacy of a 1% Metformin Chip as an Adjunct to Nonsurgical Periodontal Therapy: A Randomized, Double-Blind, Placebo-Controlled Clinical Trial

**DOI:** 10.7759/cureus.84248

**Published:** 2025-05-16

**Authors:** Dakavaram soundarya, Sumanth Gunupati, Sukrutha Biradavolu, Sreenivas Nagarakanti, Sravya sri Prudhvi, Dasari Vanditha

**Affiliations:** 1 Department of Periodontology, Narayana Dental College and Hospital, Nellore, IND

**Keywords:** local drug delivery, metformin, periodontitis, porphyromonas gingivalis, scaling and root planing (srp)

## Abstract

Objective: This study aims to evaluate the clinical and microbiological effectiveness of a 1% metformin biodegradable chip as an adjunct to scaling and root planing (SRP) in patients diagnosed with stage II, grade B periodontitis.

Materials and methods: A randomized, double-blind, placebo-controlled, parallel-group, single-center trial was conducted in systemically healthy patients diagnosed with stage II, grade B periodontitis. Patients were randomly assigned to two groups: the test group (SRP + 1% metformin chip) and the control group (SRP + placebo chip). Clinical and microbiological parameters were evaluated at baseline and three months post-treatment. These included plaque index (PI), modified sulcus bleeding index (mSBI), probing pocket depth (PPD), clinical attachment level (CAL), and microbial load, which was assessed by quantifying the colony-forming units (CFUs) of *Porphyromonas gingivalis.*

Results: Significant improvements in PI and mSBI scores were observed in both groups at three months. However, the test group exhibited a more substantial reduction in PI (0.56 ± 0.58 vs. 1.00 ± 0.58, p = 0.03) and mSBI (1.52 ± 0.51 vs. 1.00 ± 0.65, p = 0.012). While the PPD reduction was not statistically significant (p = 0.24), CAL showed significant improvements (p = 0.001), with the test group demonstrating better outcomes. Microbiological analysis revealed a significant reduction in *P. gingivalis* CFUs in both groups, with a greater bacterial reduction observed in the test group (p = 0.006).

Conclusion: The adjunctive use of a 1% metformin chip with SRP significantly enhances clinical outcomes and reduces microbial load, supporting its potential as an effective therapeutic approach for the management of periodontitis.

## Introduction

Periodontal disease is a chronic inflammatory condition affecting the tooth-supporting structures, primarily caused by the accumulation of plaque microflora and dental calculus. This microbial challenge elicits a host immune response characterized by the release of proinflammatory cytokines, which contributes to disease progression [1]. The subgingival plaque in periodontal disease is predominantly anaerobic bacteria, with 90% of the bacterial population consisting of anaerobes, of which 75% are gram-negative species. Key pathogens include *Treponema *species, *Bacteroides forsythus*, and *Porphyromonas gingivalis* [2]. The primary objective of both nonsurgical and surgical periodontal treatments is to restore periodontal tissue health by eliminating infected pockets and disrupting the dysbiotic microbial environment, thereby halting disease progression [3].

Scaling and root planing (SRP) is widely regarded as the gold standard for nonsurgical periodontal therapy. However, its effectiveness may be limited in severe periodontal infections due to the anatomical complexity of root structures [4]. While SRP significantly reduces the subgingival bacterial load, it may not fully eliminate all periodontal pathogens. In cases of moderate-to-advanced periodontal disease, adjunctive antimicrobial treatments, including antibiotics and antiseptics, are commonly used. However, systemic antibiotics often require higher doses to achieve effective concentrations at the site of infection, which may increase the risk of systemic side effects [5].

Local drug delivery systems are designed to specifically target periodontal pathogens within periodontal pockets and are available in various formulations, including chips, gels, fibers, strips, microspheres, and nanospheres. Commonly used antimicrobial agents include chlorhexidine chips, azithromycin gel, tetracycline fibers, and minocycline microspheres [6]. When placed in periodontal pockets, these delivery systems can achieve local drug concentrations 10-100 times higher than those attained through systemic administration, with minimal or no systemic side effects. Due to the limitations associated with systemic chemotherapy, local drug delivery devices were developed [7].

Metformin, a second-generation biguanide, is widely prescribed as an oral antidiabetic agent for the management of type 2 diabetes mellitus. Its primary mechanism of action involves the inhibition of hepatic gluconeogenesis and the enhancement of peripheral insulin sensitivity, resulting in lower blood glucose levels [8]. In addition to its glycemic effects, metformin offers several ancillary benefits, including modest weight loss, reduced plasma lipids, and potential anti-atherogenic and anti-cancer properties. It is also prescribed for the treatment of polycystic ovary syndrome (PCOS). Research has indicated that metformin may lower fracture risk in diabetic patients and may play a role in managing kidney disease [9].

Recent in vitro studies have demonstrated that metformin promotes osteoblastic activity by activating alkaline phosphatase and enhancing type I collagen synthesis, thereby supporting osteoblastic development. Metformin modulates host immune responses by reducing the production of pro-inflammatory cytokines such as TNF-α, IL-1β, and IL-6, contributing to the control of chronic inflammation. It further regulates immune cell function by promoting anti-inflammatory M2 macrophages and regulatory T cells while suppressing pro-inflammatory M1 macrophages and Th17 cells. Through the activation of AMP-activated protein kinase (AMPK), metformin mitigates oxidative stress and downregulates inflammatory signaling pathways. Additionally, by limiting the production of reactive oxygen species (ROS), metformin protects tissues from immune-mediated damage, creating an environment conducive for tissue repair and regeneration, particularly within inflamed periodontal tissues [10].

Studies evaluating various concentrations of metformin gel (0.5%, 1%, and 1.5%) for the treatment of chronic periodontitis have shown that 1% metformin gel is the most effective, demonstrating significant improvements in both clinical and microbiological outcomes [11].

To enhance delivery and prolong therapeutic effects, biodegradable drug delivery systems have been developed in the form of chips containing 1% metformin. These chips are fabricated using a polyethylcellulose membrane and are designed for targeted, sustained drug release directly into periodontal pockets. Once inserted, the chip gradually degrades, releasing over time at the site of infection. This localized, sustained-release approach aims to maximize therapeutic efficacy while minimizing systemic exposure, thereby improving clinical outcomes in the management of periodontitis.

The null hypothesis (H₀) of this study is that there is no significant difference in clinical and microbiological outcomes between SRP alone and SRP combined with the local delivery of a 1% biodegradable metformin chip in patients with stage II, grade B periodontitis.

Accordingly, this study aims to evaluate the clinical and microbiological effectiveness of locally administered biodegradable 1% metformin chips as an adjunct to SRP in patients with stage II, grade B periodontitis. The primary focus is on assessing equivalence in mean changes of probing pocket depth (PPD) and clinical attachment level (CAL), while also examining differences in other clinical parameters and microbial responses, with particular emphasis on *P. gingivalis*, a major periodontal pathogen.

## Materials and methods

Study design

This study aimed to investigate the clinical and microbiological effectiveness of 1% metformin biodegradable chips, applied directly at the site as an adjunct to SRP, for treating stage II, grade B periodontitis. The primary outcomes focused on equivalence, with secondary outcomes assessing potential superiority. Conducted at the Department of Periodontology, Narayana Dental College and Hospital, Nellore, India, this single-center trial employed a split-mouth, placebo-controlled, double-blind, parallel-arm, randomized clinical design. The study was prospectively registered with the Clinical Trials Registry of India (CTRI/2022/04/042317) and adhered to the Helsinki Declaration of 1975, revised in 2013. Ethical approval was granted by the Institutional Ethical Committee (IECC/NDCH/2022/Mar/P-03).

The objectives of this study are to evaluate the clinical efficacy of a 1% subgingival metformin chip compared to a placebo chip in the management of stage II, grade B periodontitis, to compare therapeutic outcomes between the two groups, and to assess differences in microbiological parameters between the test and control groups.

Study population and recruitment

Participants were recruited from the outpatient Department of Periodontology at Narayana Dental College and Hospital, India. Written informed consent was obtained from all participants after a thorough explanation of the study objectives and expected outcomes. The study was conducted from June 2022 to July 2023. Eligibility criteria were applied to include only suitable candidates.

Inclusion criteria comprised systemically healthy individuals with periodontal sites exhibiting PPD >3 mm and ≤5 mm and CAL 3-4 mm. Exclusion criteria included patients with known systemic diseases, those currently using systemic metformin or other antidiabetic treatments, tobacco users, immunocompromised individuals, pregnant or lactating women, and patients who underwent any form of periodontal therapy within the previous six months.

Sample size, randomization, and allocation

An a priori sample size calculation was made performed based on clinical parameters. Using power analysis with an effect size of 0.86, a significance level (α) of 1.96, and a β value of 1.03, the study power was set at 85%. The required sample size was 50 sites, with 25 sites per group. Accounting for potential dropouts, a total of 29 participants were planned for recruitment.

**Figure 1:**
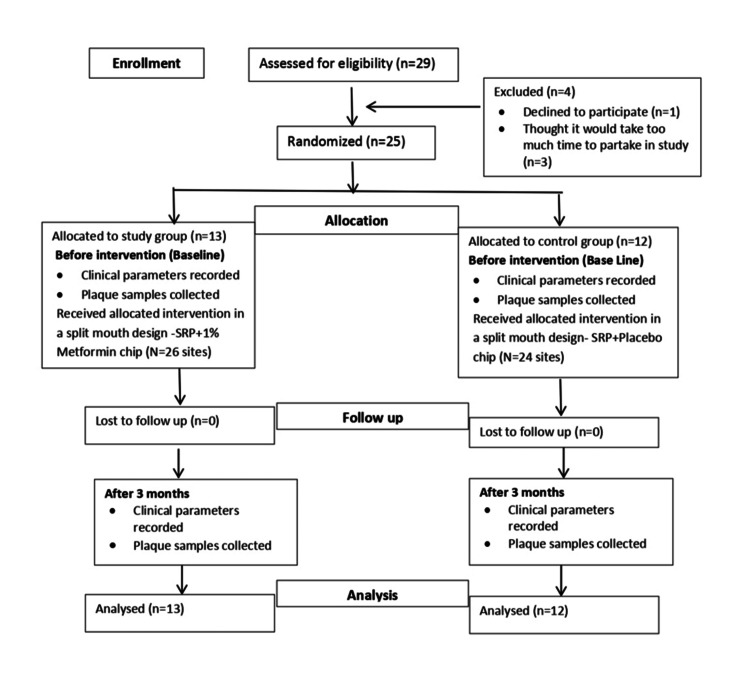
CONSORT flowchart "N" represents the number of sites; "n" represents the number of participants. SRP: scaling and root planing.

The participants were screened for eligibility. A total of 25 patients, aged 30-60 years with stage II, grade B periodontitis, were enrolled after baseline examination (Figure 1). Participants were randomly assigned to either the test group (SRP + 1% metformin chip) or the control group (SRP + placebo chip) using a computer-generated randomization chart. Blinding was maintained by using identical containers for both the metformin and placebo chips, concealing group allocation.

Intervention

Clinical parameters assessed included the plaque index (PI), modified sulcus bleeding index (mSBI), PPD, and CAL. These measurements were recorded at baseline and three months post-treatment. To ensure measurement consistency, an acrylic stent was used for standardized probing.

The stent was fabricated by taking alginate impressions of the maxillary and mandibular arches, which were then cast using dental stone. Cold-cure acrylic resin was used to create stents that fit over the occlusal third of the teeth. A groove was incorporated into each stent to guide standardized probe entry at different time points.

The study group consisted of 26 sites treated with SRP followed by local delivery of the 1% metformin chip, while the control group included 24 sites treated with SRP followed by the delivery of a placebo chip. Subgingival plaque samples were collected using a Gracey curette prior to the intervention in both groups. A split-mouth design was implemented in each group, with two sites per patient exhibiting baseline PPDs as per the inclusion criteria. The sites were labelled as site 1 (S1) and site 2 (S2), resulting in a total of 50 sites from 25 patients.

Site-specific drug delivery

After completing SRP, a 1% metformin chip was carefully inserted into the residual periodontal pocket, ensuring it reached the full pocket depth to maximize direct contact with the affected tissues. This approach aimed to provide sustained release of metformin, enhancing therapeutic effects at the site of inflammation. To stabilize the chip and protect the area from external disturbance, a periodontal dressing was applied.

Post-procedural instructions

Patients were given detailed and specific post-treatment instructions, including avoiding chewing on the treated side and refraining from touching or manipulating the site with the tongue, fingers, floss, or toothpicks. These guidelines were intended to support optimal healing and maximize the effectiveness of the metformin chip therapy.

Metformin chip

Alternatives to the metformin chip for local drug delivery in periodontal therapy include various formulations and delivery systems designed to provide sustained release and targeted therapeutic effects. These alternatives can vary in material, form, and release mechanisms: gels, nanoparticles, films or strips, microspheres or microcapsules, hydrogel systems, and electrospun fibers.

The study is unique because metformin, when applied locally, exhibits anti-inflammatory, antimicrobial, and tissue-regenerative properties that help reduce bacterial load, control inflammation, and promote tissue healing. The metformin chip was selected for this study due to its distinct advantages over other local drug delivery methods. As a biodegradable insert, the chip enables sustained and targeted release of metformin directly into the periodontal pocket, maintaining consistent therapeutic levels at the site of inflammation without the need for repeated application. This localized approach enhances the drug’s anti-inflammatory, antimicrobial, and regenerative effects while minimizing systemic exposure. Unlike gels or rinses that can be easily displaced or diluted by saliva and crevicular fluid, the chip remains in place, ensuring prolonged contact with the affected tissues. Its biodegradability eliminates the need for removal, improving patient comfort and reducing clinical follow-up. Additionally, it minimizes reliance on patient compliance, making it a more controlled and predictable treatment modality. These features make the metformin chip a clinically effective and practical choice for enhancing outcomes in the management of periodontitis.

Preparation of metformin chip

The materials used in this study included 2.5 mg of metformin, formulated into a 1% metformin solution. This solution was incorporated into a polyethylcellulose membrane developed with the assistance of PerioBiologics LLP (Hyderabad, India). Each metformin chip measured 6 x 7 mm, with a thickness of 1.3 mm and a weight of approximately 23 mg.

The polyethylcellulose membrane was prepared by first combining sodium alginate and water to form a gel mixture. This mixture was triturated using an overhead mechanical stirrer at 400 rpm on a hotplate set at 70 °C, and then cooled to 30-40 °C. Calcium carbonate was subsequently added as a cross-linking agent. Sodium carbonate and metformin were then incorporated to complete the chip formulation. Hexaethyl cellulose was included as a thickening agent while preparing a 1% metformin chip suitable for local drug delivery. The entire preparation process was conducted under aseptic conditions within a cleanroom environment. The cellulose-metformin chip was cross-linked with calcium carbonate to enhance its in vivo persistence. The cross-linking density was carefully controlled to achieve the desired degradation rate. Each chip was individually packed in sterile pouches and sterilized using ethylene oxide. The placebo chip was prepared following the same protocol, except that it did not contain the 1% metformin.

Intraoral microbiological outcomes

The test and control sites were isolated, and subgingival plaque samples were collected from both groups prior to SRP. Plaque was obtained using a Gracey curette and transferred into Eppendorf tubes containing Tris-EDTA medium. The samples were then transported to the microbiological lab in an icebox for further analysis. Samples were cultured on modified Wilkins-Chalgren (MWC) medium to detect colony-forming units (CFUs) of *P. gingivalis* only. Bacterial colony growth was monitored at specific time intervals, with digital photographs captured every 24 hours for morphometric analysis using the ImageJ software plugin.

*P. gingivalis* (ATCC 33277) was procured from the American Type Culture Collection (ATCC, USA), and the bacteria were cultured under anaerobic conditions consisting of 85% nitrogen (N_2_), 10% hydrogen (H_2_), and 5% carbon dioxide (CO_2_). A 4-mL aliquot of the liquid culture was transferred to a test tube, and a single colony was incubated at 37 °C for 16-24 hours. A portion of the culture medium was preserved at -80 °C for future use. Bacterial density was standardized using the McFarland standard turbidity method. The turbidity of each culture was adjusted to an absorbance range of 0.09-0.13, corresponding to a density of 1 × 10^7^ CFU/mL, using McFarland Turbidity Standard No. 0.5 kit (bioMérieux, France). Absorbance measurements were taken at 600 nm, with a 6131 BioPhotometer (Eppendorf, Germany). All microbial analyses were conducted at SVSES Microbiological Services, Mahbubnagar 509002, Telangana, India.

Statistical analysis

The collected data were statistically analyzed using IBM SPSS Statistics for Windows, Version 21.0 (Released 2012; IBM Corp., Armonk, NY, USA). For intragroup comparisons of quantitative clinical variables, both the unpaired t-test and the Mann-Whitney U test were employed, depending on the distribution of the data. Microbiological comparisons between groups were analyzed using the Mann-Whitney U test due to nonparametric data distribution. Intergroup comparisons of clinical and microbiological outcomes were assessed using mean difference scores. A p-value < 0.05 was considered statistically significant.

## Results

The clinical trial included 13 women (mean age: 42.54 ± 5.39 years) and 12 men (mean age: 37.33 ± 6.30 years). Clinical parameters were recorded at baseline and three months post-treatment for both the control and test groups. Significant improvements were observed in both groups, with the test group demonstrating more notable enhancements.

The normality of the data for clinical parameters at both time points was assessed using the Shapiro-Wilk test. As the data did not follow a normal distribution, nonparametric statistical methods were employed for further analysis.

Based on the results, statistically significant improvements were observed in the test group compared to the control group. Therefore, the null hypothesis was rejected, indicating that the adjunctive use of a 1% metformin chip provides a beneficial effect on both clinical and microbiological outcomes in the management of stage II, grade B periodontitis.

The intergroup comparison of clinical and microbiological parameters between the control and test groups at baseline and three months post-treatment is presented in Table 1. At baseline, the PI was 2.08 ± 0.81 in the control group and 2.28 ± 0.68 in the test group. After three months, the PI scores decreased to 1.52 ± 0.59 in the control group and 1.28 ± 0.54 in the test group. The reduction in PI scores was statistically significant, with a difference of 0.56 ± 0.58 in the control group and 1.00 ± 0.58 in the test group (p = 0.03). The mSBI score at baseline was 2.28 ± 0.68 for both the test and control groups. After three months, the mSBI score decreased to 1.52 ± 0.51 in the control group and 1.00 ± 0.65 in the test group. This reduction was statistically significant, with a p-value of 0.012. The PPD scores at baseline were 6.16 ± 0.80 for the control group and 6.04 ± 0.79 for the test group. At the three-month follow-up, the PPD scores reduced to 4.56 ± 0.88 for the control group and 4.20 ± 0.91 for the test group. However, the difference was not statistically significant, with a p-value of 0.24. The CAL at baseline was 6.16 ± 0.80 for the control group and 6.00 ± 0.71 for the test group. At the three-month follow-up, the CAL scores decreased to 5.12 ± 0.78 for the control group and 3.76 ± 0.60 for the test group. The difference observed was statistically significant, with a p-value of 0.001. The CFUs of *P. gingivalis* at baseline were 594.84 ± 53.09 for the control group and 573.76 ± 57.32 for the test group. At the three-month follow-up, the CFU counts decreased to 565.92 ± 57.79 for the control group and 525.08 ± 53.67 for the test group. This reduction was statistically significant, with a p-value of 0.006.

**Table 1:** Intergroup comparison of clinical and microbiological parameters of control and test sites at baseline and three months post-treatment *Statistically significant (p < 0.05). Mann-Whitney U test used. SD: standard deviation, Z-value: standard score, mSBI: modified sulcus bleeding index, PPD: probing pocket depth, CAL: clinical attachment level, CFUs: colony-forming units.

Parameters	Time points	Control site		Test site		Z-value	p-value
		Mean	SD	Mean	SD		
PI	Baseline	2.08	0.81	2.28	0.68	0.7858	0.4320
	Three months	1.52	0.59	1.28	0.54	1.3873	0.1654
	Difference	0.56	0.58	1.00	0.58	2.0664	0.0388*
mSBI	Baseline	2.28	0.68	2.28	0.68	0.0097	0.9923
	Three months	1.52	0.51	1.00	0.65	2.5127	0.0120*
	Difference	0.76	0.44	1.28	0.46	-2.7358	0.0062*
PPD	Baseline	6.16	0.80	6.04	0.79	0.5045	0.6139
	Three months	4.56	0.82	4.20	0.91	1.1739	0.2404
	Difference	1.60	0.71	1.84	1.18	-0.1261	0.8996
CAL	Baseline	6.16	0.80	6.00	0.71	0.7276	0.4669
	Three months	5.12	0.78	3.76	0.60	4.7537	0.0001*
	Difference	1.04	0.20	2.24	0.97	-4.4433	0.0001*
CFUs of *P. ginvalis*	Baseline	594.84	53.09	573.76	57.32	1.1836	0.2366
	Three months	565.92	57.79	525.08	53.67	2.7455	0.0060*
	Difference	28.92	17.29	48.68	13.46	-4.0649	0.0001*

The intragroup comparison of changes in clinical and microbiological parameters showed a statistical significance (p < 0.01) for both the control and test groups from baseline to three months (Table 2).

**Table 2:** Intragroup comparison of all parameter scores at baseline and three months post-treatment *Statistically significant (p < 0.05). Wilcoxon matched pairs test used. Z-value: standard score, mSBI: modified sulcus bleeding index, PPD: probing pocket depth, CAL: clinical attachment level, CFU: colony-forming unit.

Parameter	Groups	Changes from	Mean change	% of change	Z-value	p-value
PI	Control group	Baseline to three months	0.56	26.92	3.0767	0.0021*
	Test group	Baseline to three months	1.00	43.86	4.0148	0.0001*
mSBI	Control group	Baseline to three months	0.76	33.33	3.8230	0.0001*
	Test group	Baseline to three months	1.28	56.14	4.3729	0.0001*
PPD	Control group	Baseline to three months	1.60	25.97	4.3724	0.0001*
	Test group	Baseline to three months	1.84	30.46	4.3725	0.0001*
CAL	Control group	Baseline to three months	1.04	16.88	4.3712	0.0001*
	Test group	Baseline to three months	2.24	37.33	4.3724	0.0001*
CFU	Control group	Baseline to three months	28.92	4.86	4.3724	0.0001*
	Test group	Baseline to three months	48.68	8.48	4.3730	0.0001*

## Discussion

Periodontitis is a chronic inflammatory disease affecting the tooth-supporting apparatus, including the gingiva, periodontal ligament, cementum, and alveolar bone. It typically arises from untreated gingivitis and is characterized by the progressive destruction of these supporting tissues, leading to the formation of periodontal pockets, alveolar bone loss, tooth mobility, and eventual tooth loss. Dental plaque, a biofilm of pathogenic organisms, is the primary cause of periodontitis. The goal of periodontal therapy is to eliminate both supra- and subgingival plaque biofilms. Mechanical debridement is the gold standard, and the use of adjunctive treatments can further enhance the clinical outcomes [6].

Metformin, an antidiabetic agent, has been explored for the treatment of periodontitis due to its multiple beneficial properties, including direct anti-inflammatory effects [12], osteogenic activity on osteoblasts [13], inhibition of osteoclast function, antimicrobial effects [14], antioxidant properties, and its potential in mitigating atherosclerosis, obesity, and tumors. Additionally, metformin is a cost-effective agent, derived from renewable plant-based resources, and has demonstrated potential for use in various inflammatory conditions, including chronic periodontitis, when administered as a local drug delivery system [8]. Multiple studies have demonstrated that metformin application improves both clinical and radiographic outcomes following SRP [9,10,12].

PerioChip, a commercially available, FDA-approved local drug delivery agent containing 0.02% chlorhexidine, is considered the gold standard for treating periodontitis [15]. However, its high cost is a limiting factor. Subgingival usage of metformin in periodontal therapy offers several therapeutic advantages that enhance both clinical and microbiological outcomes. Its anti-inflammatory properties help lower the levels of pro-inflammatory cytokines such as TNF-α, IL-1β, and IL-6, which are crucial in driving tissue damage in periodontitis. Metformin also exhibits antimicrobial activity, particularly against periodontal pathogens like *P. gingivalis*, helping reduce bacterial load in the subgingival biofilm and manage infection.

In terms of CAL, metformin aids tissue regeneration by stimulating the proliferation of periodontal ligament cells and promoting osteoblast differentiation, which leads to reattachment of the tissues to the tooth. Furthermore, its osteoprotective effects reduce bone resorption by inhibiting osteoclasts and enhancing osteoblast function, thereby supporting bone regeneration in the affected areas. By activating the AMPK pathway, metformin improves cellular metabolism, reduces oxidative stress, and creates a conducive environment for tissue repair. When delivered via a biodegradable chip, metformin provides a sustained release directly into the periodontal pocket, ensuring prolonged therapeutic effects that support healing, control inflammation, and reduce microbial load over time [10,11].

To overcome this, we used an indigenously developed biodegradable chip, offering a more affordable alternative in the treatment of patients with stage II, grade B periodontitis. Thus, the present study aims to compare the effectiveness of SRP with local delivery of biodegradable 1% metformin chip (test group) versus SRP with a placebo chip (control group).

A marked reduction in the PI from baseline to three months was observed, consistent with previous studies highlighting the effectiveness of SRP in controlling dental plaque accumulation. Notably, the greater reduction in PI in the test group suggests that the adjunctive use of metformin may further enhance plaque control. As PI reflects patients’ oral hygiene maintenance, its reduction also indicates better periodontal health. Metformin, due to its antimicrobial activity against key periodontal pathogens, helps reduce bacterial colonization and biofilm formation. Furthermore, it suppresses inflammation by downregulating pro-inflammatory cytokines and modulates the host immune response through activation of the AMPK pathway. These effects collectively create a tissue environment less favorable for bacterial proliferation and more conducive to healing. These findings are in line with Patil et al. [16], who reported that metformin significantly reduces dental plaque and improves periodontal health outcomes [17].

The clinical parameter mSBI showed a significant reduction in both groups, with the test group demonstrating more substantial improvement. This suggests a notable reduction in gingival inflammation, a critical factor in the management of periodontal disease. The anti-inflammatory effects of 1% metformin, as highlighted by Uddin et al. [18], support its role in enhancing periodontal health [19]. However, in contrast to the findings of Pradeep et al. [17], our study did not demonstrate a statistically significant improvement in PPD with the use of the 1% metformin chip. Although the reduction in PPD was not statistically significant between the groups, the observed numerical improvement remains clinically meaningful. This lack of statistical significance may be attributed to factors such as a relatively small sample size, short follow-up period, or individual variability in healing responses, all of which may attenuate detectable differences in clinical trials.

The statistically significant intragroup reduction in PPD observed in the metformin-treated group is clinically meaningful, as it indicates a genuine therapeutic benefit over time within this group. A decrease in PPD is associated with reduced periodontal inflammation, improved gingival and connective tissue health, and a diminished risk of disease progression. This improvement suggests that metformin contributes positively to periodontal pocket healing, likely due to its combined anti-inflammatory, antimicrobial, and regenerative properties. Such effects can facilitate better long-term periodontal maintenance, enhance patient's ability to control plaque, and improve overall clinical outcomes. These findings support the potential of 1% metformin as a valuable adjunct in nonsurgical periodontal therapy.

However, CAL remains a key indicator in evaluating the success of periodontal therapy, as it reflects true periodontal tissue regeneration and healing. In the present study, both the control and test groups demonstrated significant improvements in CAL, underscoring the efficacy of SRP in controlling periodontal disease and promoting tissue reattachment. However, the test group receiving the 1% metformin chip showed notably greater improvements in CAL. This enhancement may be attributed to metformin’s multifaceted regenerative mechanisms. Metformin promotes the proliferation and differentiation of periodontal ligament cells and osteoblasts, contributing to tissue regeneration. Its anti-inflammatory effects help mitigate tissue destruction, while its inhibition of osteoclast activity supports bone preservation and formation. Furthermore, metformin stimulates the expression of VEGF, facilitating angiogenesis and tissue repair. These regenerative actions are mediated in part by activation of the AMPK pathway, which enhances cellular energy homeostasis and supports periodontal healing. These findings are consistent with those of Rao et al. [20], who reported significant CAL gains with the use of 1% metformin in patients with chronic periodontitis [10,11,21].

The microbiological analysis focused on quantifying CFUs of *P. gingivalis*, one of the key periodontal pathogen. Both groups demonstrated a significant reduction in CFUs of *P. gingivalis* after three months. However, the test group showed a more pronounced reduction compared to the control group.

These results underscore the effectiveness of SRP in mechanically reducing bacterial load within periodontal pockets. The greater reduction observed in the test group suggests that 1% metformin may exert an additional antimicrobial effect, potentially by enhancing host immunity rather than directly eliminating the pathogen [22].

In this study, a direct subgingival delivery method was used to deliver a 1% metformin chip into the periodontal pockets of patients with stage II, grade B periodontitis. Local drug delivery systems offer several advantages, such as higher drug concentrations at the target site, lower dosages, fewer applications, and better patient acceptance. Compared to systemic treatments, local delivery significantly reduces adverse reactions and improves patient compliance.

Study limitations and future directions

Although this study provides valuable insights, it is not without limitations. The relatively small sample size and brief follow-up period may restrict the generalizability of the findings. To strengthen the evidence, future research should involve larger sample sizes and multicenter cohorts with extended follow-up periods to further validate these results and assess the long-term sustainability of the clinical and microbiological improvements. Furthermore, the study could have been more unique and impactful if it had included comparisons with other drugs or treatment modalities, which would have provided a broader context for evaluating metformin's effectiveness. The lack of such comparative analysis limits the ability to fully assess metformin's relative advantages and efficacy in periodontal care. This study focused on analyzing a single microorganism (*P. gingivalis*), which does not fully represent the complex microbial ecosystem involved in periodontal disease. Future research could incorporate broader molecular techniques, such as qPCR or 16S rRNA sequencing, to provide a more comprehensive understanding of the microbial diversity associated with periodontal conditions.

As a 1% metformin chip is not yet commercially available worldwide, this study serves as a foundational research model until it becomes widely accessible. Furthermore, additional investigations are necessary to better understand the mechanisms driving the positive outcomes of metformin in periodontal treatment.

## Conclusions

This study demonstrates that the local administration of a biodegradable 1% metformin chip, when used as an adjunct to SRP, enhances both clinical and microbiological outcomes in patients with stage II, grade B periodontitis. The findings underscore the effectiveness of 1% metformin in controlling dental plaque, minimizing inflammation, and promoting the healing of periodontal tissues. The innovative use of a biodegradable 1% metformin chip ensures sustained drug release and prolonged therapeutic effects, offering a cost-effective and promising enhancement in periodontal treatment approaches.
